# Septic Lateral Sinus Thrombosis: Sinus Exploration Is Unnecessary

**DOI:** 10.1155/2016/4349538

**Published:** 2016-01-12

**Authors:** Gautam Bir Singh, Rubeena Arora, Sunil Garg, Deepak Kumar, Shruti Ranjan

**Affiliations:** ^1^Department of Otorhinolaryngology, Head & Neck Surgery, Lady Hardinge Medical College & Associated Hospitals, Shaheed Bhagat Singh Marg, New Delhi 110001, India; ^2^Department of Otorhinolaryngology, Head & Neck Surgery, Dr. Baba Saheb Ambedkar Medical College & Hospital, Rohini, New Delhi 110085, India; ^3^Department of Otorhinolaryngology, Head & Neck Surgery, ESI Hospital, Rohini, New Delhi 110085, India

## Abstract

The algorithm of treatment of septic lateral sinus thrombosis (SLST) has undergone a paradigm shift with the understanding of the natural history of sigmoid sinus thrombosis. Thus, the recent medical literature promulgates the management of these cases with no sinus exploration. However, in view of marked paucity of literature on the cited subject, not much is known about this form of treatment. We present our experience of treating two paediatric cases of SLST with mastoid surgery and no sinus exploration: both cases had excellent recovery. Finally, conclusions are drawn in light of contemporary literature on this subject.

## 1. Introduction

Septic lateral sinus thrombosis (SLST) is a rare complication of chronic suppurative otitis media (CSOM) in paediatric population (accounts for 2–20% of all intracranial complications) [1]. Interestingly, the incidence of SLST has increased with the advancement in medical diagnostics. However, early diagnosis and use of potent antibiotics have markedly decreased the mortality to less than 5% [1]. In this context, it would be important to note that the clinical presentation of SLST has become more subtle with the advent of potent and new antibiotics [1, 2]. The complication no longer presents with classical clinical features mentioned in the medical text, severe wasting illness with “Picket Fence” fever.

Management of SLST varies considerably in the literature. The traditional teaching has been to do a mastoid surgery with sinus exploration under antibiotic cover [1–3]. Some suggest incision of the sinus with evacuation of the clot, while others have adopted a more conservative approach: needle aspiration of the sinus. There is also no consensus among otologists on the role of anticoagulants and ligation of internal jugular vein [2, 3]. Recent review of literature mentions the management of SLST with no sinus exploration [1–3]. Ideally, the effect of variation in sinus management on morbidity and mortality of SLST should be studied by a randomized control trial. However, given the limited number of cases of SLST such an endeavour may be difficult to achieve. With this background we present our modest experience of two cases of SLST which were managed by mastoid surgery only and no sinus exploration.

## 2. Case I

**Figure 1:**
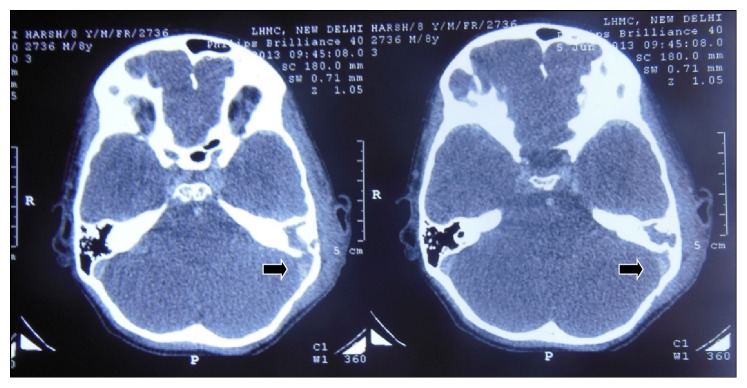
Contrast-Enhanced CT scan showing lateral sinus thrombosis (arrow).

**Figure 2:**
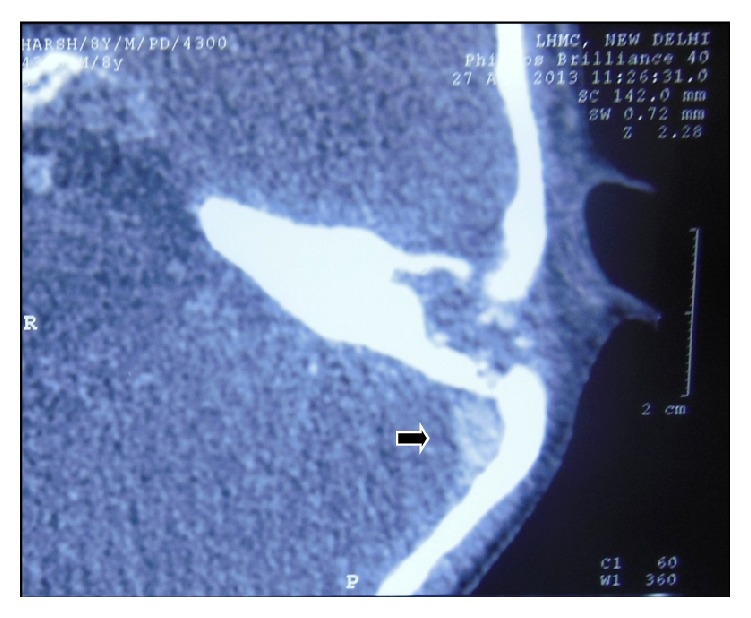
Contrast-Enhanced CT scan showing recanalized left sigmoid sinus (arrow).

An 8-year-old boy reported to the ENT Emergency with chief complaints of ear discharge and pain in the left ear with postauricular swelling. Patient also had a history of fever, headache, and nausea and vomiting. The patient had been suffering from CSOM left ear for the past 3 years and had been taking antibiotics for the same off and on. However, no medical records were available for the antibiotic treatment. Examination of the ear showed a perforation in pars flaccida, erosion of scutum with foul smelling discharge, and cholesteatoma flakes. In the postauricular region 4 × 2 cm boggy inflammatory swelling was also seen, which had pus on needle aspiration. In view of intracranial symptoms a medical opinion was sought and subsequent CECT scan (Contrast-Enhanced Computed Tomography) revealed lateral sinus thrombosis (Figure 1). Hence, a final diagnosis of CSOM left ear-cholesteatoma disease with postauricular abscess and lateral sinus thrombosis was made. Patient underwent modified radical mastoidectomy with drainage of the abscess (but no sinus exploration) under antibiotic cover. Patient recovered satisfactorily with no untoward incident to report and was discharged subsequently at the end of 3 weeks. A repeat CECT scan done at the end of 8 weeks was normal showing recanalization of left sigmoid sinus (Figure 2).

*Surgical Pathology*

*Postauricular Abscess*. 5 mL pus was drained.

*Cholesteatoma*. Cholesteatoma was seen in aditus, antrum, attic, epitympanum, PSQ of tympanic cavity involving facial recess, and sinus tympani.

*Ossicles*. Status of ossicles: long process of incus, handle of malleus, and stapes suprastructure were necrosed.

## 3. Case II

**Figure 3:**
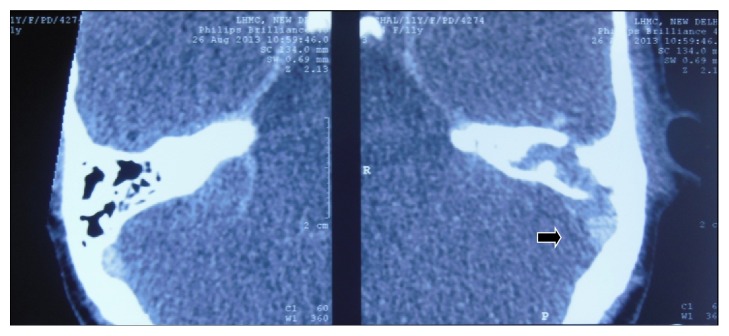
Contrast-enhanced CT scan showing mastoiditis and lateral sinus thrombosis (arrow).

An 11-year-old girl reported to the ENT OPD with the complaint of persistently discharging left ear for the past 3 months. In addition, for the past 10 days patient had also developed ear pain and persistent headache. However, patient had no other clinical features of raised intracranial tension. Past history revealed that this girl had been suffering from CSOM left ear for the last 5 years with gradually deteriorating hearing loss. History of rampant use of antibiotics with no medical records was also present. Examination of the ear revealed posterosuperior perforation in the tympanic membrane (involving both pars tensa and the adjoining pars flaccida) with granulations and cholesteatoma flakes. The Griesinger sign was positive. A CT scan of this patient revealed destructive bony changes with dilated sigmoid sinus with partial hypodense filling defect on left side: lateral sinus thrombosis (Figure 3). Thus, a diagnosis of CSOM left ear-cholesteatoma disease with lateral sinus thrombosis was made. An urgent modified radical mastoidectomy (no sinus exploration) was under antibiotic cover. The patient was discharged at the end of 3 weeks with excellent prognosis.

*Surgical Pathology*

*Granulations with Cholesteatoma*. Antrum, aditus, attic, and posterosuperior quadrant of tympanic cavity involved facial recess and sinus tympani.

*Ossicles*. Long process of incus and stapes suprastructure were necrosed.

It would be prudent to note that “ear swabs” were sterile and “hypercoagulability” was absent in both cases. Both patients were treated initially with a combination of amoxicillin and clavulanic acid and metronidazole intravenously for duration of 2 weeks, followed by oral antibiotics for the next two weeks.

## 4. Discussion

Some clinical observations regarding these cases merit discussion. Both of our cases had a history of protracted CSOM with abuse of antibiotics. The medical literature cites antibiotic resistance as an important cause for aetiopathogenesis of SLST [2]. Further, both the cases had an actively discharging ear at the time of diagnosis of this complication, thereby implying that “Acute Suppurative Otitis Media (ASOM)” is an important predisposing factor. Several studies have also delineated the role of ASOM in causing intracranial complications [2]. Both cases had an atypical clinical presentation and were diagnosed on CT scan. Although literature is replete with references that suggest “hypercoagulability” as an important predisposing factor for OLST [2, 4], no “hypercoagulability” state was detected in any of our patients.

Although we diagnosed these cases by CT scan, “Magnetic Radio Imaging (MRI)” is regarded as a more sensitive investigation to diagnose SLST. However, this should be performed in conjunction with CT scan to evaluate associated otologic and cerebral pathology [3, 5]. Despite the advantages of MRI, its cost and selective availability, especially in developing countries, limit its use and it is thus mandatory only in those suspicious cases where CT scan fails to demonstrate the thrombus. This view has been endorsed by other otologists too [1, 2, 6]. We believe that CT scan provides useful corollary information. It would however be pertinent to note that MR venography/arteriography now supersedes all other investigations for the identification of the thrombus in the sigmoid sinus as evidenced by flow void [1, 2].

There is considerable controversy regarding surgical management of SLST: the role of mastoid surgery is also not fully characterized. Further, there is marked surgical dilemma regarding the involved thrombosed sinus: many advocate incision of sinus with removal of the clot, while other simply unroof the sinus and confirm the presence of thrombus with needle aspiration. Both of our cases of SLST had excellent recovery after mastoid surgery only. In no case the involved sinus was explored. This treatment is based on the principle that thrombus formation is secondary to infection, and thus treatment of infection is the core management issue (not the thrombus). It is reasoned that formation of thrombus is a protective mechanism attempting to localize infection, and the natural history of SLST is of resolution. The venous occlusion resolves by 4 to 6 weeks with adequate antibiotic treatment only (no adjuvant surgery or anticoagulant therapy is required). Thus, once the source of infection is eradicated, the thrombus resolves [3, 4, 7]. However, it would be pertinent to note that the definitive treatment for CSOM-cholesteatoma disease is surgery [1, 2]. Hence, in all such cases with SLST, mastoid surgery is a must for eradication of the disease along with antibiotics.

In accordance with aforesaid principle, some otologists have also reported a favourable outcome in patients of SLST by removal of surrounding granulation tissue and inflammation around the sinus (reducing the thrombophilic nidus) with clot left untouched [8–10]. Also interestingly, there are also sporadic reports of management of SLST with antibiotics and anticoagulant therapy alone with no mastoid surgery at all in cases of CSOM-mucosal disease, especially in children [6, 11]. It would however be pertinent to note that the role of anticoagulants is contested [2–4]. A recent report by Shah et al. highlights the potential complications of anticoagulation therapy in children suffering from lateral sinus thrombosis [12]. We would thus like to emphasise the adjuvant role of antibiotics in cases of SLST. In both of our cases antibiotics were used for a long duration (up to 4 weeks) along with mastoid surgery to get good prognosis.

Given the success of mastoid surgery under antibiotic cover in both of our cases, we conclude that SLST in CSOM-cholesteatoma disease requires mastoid surgery with no sinus exploration. However, many questions regarding management of SLST still remain unanswered. We cite our modest experience in two cases as a template for future research and modification in the best interest of patient care.

In summary, we present this clinical record on account of (i) rarity of the cited complication, (ii) its unique management by excluding sinus exploration, and (iii) underreporting of this management in English medical literature, which limits conclusions to be drawn on the treatment of OLST in accordance with evidence based medicine.
